# Numerical Simulation of the Bearing Capacity of Variotropic Short Concrete Beams Reinforced with Polymer Composite Reinforcing Bars

**DOI:** 10.3390/polym14153051

**Published:** 2022-07-28

**Authors:** Alexey N. Beskopylny, Besarion Meskhi, Sergey A. Stel’makh, Evgenii M. Shcherban’, Levon R. Mailyan, Andrey Veremeenko, Vladimir Akopyan, Aleksandr V. Shilov, Andrei Chernil’nik, Nikita Beskopylny

**Affiliations:** 1 Department of Transport Systems, Faculty of Roads and Transport Systems, Don State Technical University, Gagarin, 1, 344003 Rostov-on-Don, Russia; 2 Department of Life Safety and Environmental Protection, Faculty of Life Safety and Environmental Engineering, Don State Technical University, Gagarin, 1, 344003 Rostov-on-Don, Russia; spu-02@donstu.ru; 3 Department of Engineering Geology, Bases, and Foundations, Don State Technical University, 344003 Rostov-on-Don, Russia; sergej.stelmax@mail.ru (S.A.S.); au-geen@mail.ru (E.M.S.); vovaakop@mail.ru (V.A.); 4 Department of Roads, Faculty of Roads and Transport Systems, Don State Technical University, 344003 Rostov-on-Don, Russia; lrm@aaanet.ru (L.R.M.); veremeenko78@mail.ru (A.V.); 5 Department of Reinforced Concrete Structures, Faculty of Industrial and Civil Engineering, Don State Technical University, Gagarin, 1, 344003 Rostov-on-Don, Russia; avshilov75@mail.ru; 6 Department of Unique Buildings and Constructions Engineering, Don State Technical University, Gagarin Sq. 1, 344003 Rostov-on-Don, Russia; chernila_a@mail.ru; 7 Department of Hardware and Software Engineering, Don State Technical University, Gagarin Sq. 1, 344003 Rostov-on-Don, Russia; beskna@yandex.ru

**Keywords:** heavy concrete, numerical simulation, bending element, variotropic structure, polymer composite reinforcement, transversely isotropic beam

## Abstract

One of the disadvantages of reinforced concrete is the large weight of structures due to the steel reinforcement. A way to overcome this issue and develop new types of reinforcing elements is by using polymer composite reinforcement, which can successfully compensate for the shortcomings of steel reinforcement. Additionally, a promising direction is the creation of variotropic (transversely isotropic) building elements. The purpose of this work was to numerically analyze improved short bending concrete elements with a variotropic structure reinforced with polymer composite rods and to determine the prospects for the further extension of the results obtained for long-span structures. Numerical models of beams of a transversally isotropic structure with various types of reinforcement have been developed in a spatially and physically nonlinear formulation in ANSYS software considering cracking and crashing. It is shown that, in combination with a stronger layer of the compressed zone of the beam, carbon composite reinforcement has advantages and provides a greater bearing capacity than glass or basalt composite. It has been proven that the use of the integral characteristics of concrete and the deflections of the elements are greater than those when using the differential characteristics of concrete along the height of the section (up to 5%). The zones of the initiation and propagation of cracks for different polymer composite reinforcements are determined. An assessment of the bearing capacity of the beam is given. A significant (up to 146%) increase in the forces in the reinforcing bars and a decrease in tensile stresses (up to 210–230%) were established during the physically non-linear operation of the concrete material. The effect of a clear redistribution of stresses is in favor of elements with a variotropic cross section in height.

## 1. Introduction

Modern construction puts forward several specific requirements for buildings and structures, as well as for the building structures, products and parts from which these buildings and structures are built. In particular, the appearance of cities is changing, the share of high-rise and large-span buildings and structures is growing, engineering and geological conditions are becoming more complicated due to the lack of territories studied from the point of view of geology and there is a need to erect buildings and structures in difficult engineering and geological conditions, as well as in conditions of dense urban development. All this contributes to the fact that modern designers and builders need to build buildings considering many complicating factors. All these problems are relevant for the construction industry, and finding ways to solve them is the main task of modern design, construction and building science. Undoubtedly, one of the most principal elements of modern construction is reinforced concrete, as well as the products, parts and structures made of it. At the same time, precast concrete is still one of the most popular types of building products due to the fact that, in the factory, it is possible to clearly control the production process. Additionally, world science in the field of building materials science is developing new ways to improve compositions, recipes, technologies, design solutions and methods for calculating and designing building elements made of heavy concrete [1,2,3,4,5,6].

It should be noted that reinforced concrete, with all its advantages, also has several disadvantages, which are expressed in the high cost of raw materials, such as reinforcing steel, and the large weight of the created structures, products and buildings [7,8,9,10,11,12]. All this leads to the fact that modern scientists and engineers are developing, with a certain degree of success, new types of reinforcing elements, such as polymer composite reinforcement, which makes it possible to successfully solve the abovementioned tasks and problems [13,14,15,16,17]. However, it should be noted that, as has already been proven by us and other authors earlier, it is certainly impossible to achieve a significant solution to the problem and eliminate all risk factors by improving the reinforcing elements alone [18,19,20,21,22,23]. Of course, such tasks should be approached in a comprehensive manner, and building elements made of heavy concrete with various reinforcing elements should be evaluated as a system operating in conditions of a certain synergy [24,25,26,27,28,29]. Such synergy, as we have already established earlier, can be complex reinforcement, namely, fiber plus reinforcing bars, improved concrete characteristics due to their nanomodification or the same fiber reinforcement with fibers [30,31,32,33,34]. However, in addition to the above methods, it is also promising to create so-called differentiated building elements, particularly in their cross section [35,36,37,38,39,40].

Relatively new for reinforcement in the calculation of building structures is high-strength non-metallic reinforcement made of composite materials [41,42,43,44,45,46]. Polymer composite reinforcement is mainly produced in the form of a rod with a spiral relief of any construction length made of glass or basalt fibers impregnated with a chemically resistant polymer [47,48,49,50,51,52,53,54,55,56,57,58,59]. Polymer composite reinforcement is characterized by good mechanical properties—namely, high tensile strength—and physical properties—for example, lower density compared to steel reinforcement [21,22,26,27,31,32,36]. Fiberglass reinforcing bars are often used in structures subjected to a strong exposure to aggressive environments. Another advantage of fiberglass reinforcement is its electromagnetic neutrality, which ensures the popularity of this type of reinforcement in the construction of infrastructure facilities with stray currents [9,14,19,21,22,47]. For CFRP, it has a higher price due to the cost of carbon fiber and the low elongation at break. However, its mechanical properties, fatigue/creep and corrosion resistances are really excellent. In contrast, the prices of GFRP and BFRP are relatively low due to the rich raw materials. However, its long-term performance exposed to loading and the environment is fragile, especially in the alkaline environment of concrete, owing to the chemical reaction between Si-O and hydroxyl ion [60,61,62].

If we talk about the nature of the destruction of reinforced concrete elements in shear, then the destruction in the shear of steel-reinforced concrete beams without transverse reinforcement often occurs suddenly and is brittle. In the case of fiberglass reinforcement, the nature of shear failure is different and is characterized by linear elastic behavior over the entire range of strength to failure. However, fiberglass reinforcing bars do not show plastic properties and break quickly. Therefore, the destruction of beams reinforced with fiberglass reinforcement during bending and shear occurs much faster than that of reinforced concrete beams. This condition indicates the importance of the process of designing bending elements reinforced with fiberglass reinforcement, considering both bending and shear. So, for example, in several works [7,8,10,16,30,45], the authors, through experimental studies and calculations, developed and proposed new recommendations for calculating the shear strength of beams longitudinally reinforced with fiberglass reinforcement. Oller at al. [30] considered a mechanical model for predicting the shear strength of beams reinforced with fiberglass reinforcement, which considers its features. The model assumed that the transverse force is taken up by a concrete belt without cracks, residual tensile stresses along the length of the crack and fiberglass staples. The obtained coefficient of variation was the lowest of all the studied methods. Thus, due to the simplicity, accuracy and mechanical construction of the model, it is suitable for design and verification in engineering practice [30].

Polymer composite reinforcement made of basalt fiber surpasses fiberglass reinforcement in its properties; however, it is inferior to carbon composite reinforcement. Additionally, basalt composite reinforcement is characterized by properties such as efficiency, resistance to high temperatures and a rather high limit of long-term strength [13,15,18,21,26,27,28,29,46,47,48,57]. The shear resistance of structural elements reinforced with basalt composite rebar is quite different from the shear response and behavior of elements reinforced with steel bars. Accordingly, the algorithm for designing structures reinforced with basalt composite reinforcement will differ significantly from the algorithm for designing reinforced concrete structures. Additionally, when designing structures with basalt composite reinforcement, the influence of the size effect is significant. A significant size effect can be seen in the shear strength of large beams with basalt composite reinforcement, even with sufficient reinforcement. Thus, the effect of size on the shear strength is an urgent problem in the design of structures made of high-strength concrete, since the total shear resistance and the height of the upper compression zone in such concretes are less than those in ordinary concrete elements [18,29,46,48]. Another actual problem in the use of reinforcement from basalt rods is the assembly of the frame. For fiberglass rebar, bending can be carried out before the rebar hardens or by heating it. The contribution of stirrups from basalt composite reinforcement to shear resistance is based on the stresses created in the stirrups, since the basalt composite rod is linearly elastic to failure, does not lend itself to deformation and has a tensile strength of the bent segment, which is much less than that of the straight part [13,18,29,46,48]. For example, in [13], the authors studied the structural characteristics of basalt composite rods in concrete beams. Beams with low reinforcement ratios showed a sharp increase in deformation and deflection during cracking. However, increasing the reinforcement ratio increased the amount of energy absorbed at the first crack, which improved the behavior, as it controls the direct increase in the strain and initial crack width [13].

Mineral impregnated carbon fiber composites are a new type of reinforcement for construction. Compared to steel reinforcement, carbon fiber reinforcement has a much higher tensile strength and a significantly lower weight [22,24,31,33,35,38,39,41,42,43,44,47,49,51,55,59]. At present, the technology of reinforcing concrete bending elements with carbon fiber sheets is popular [44,59]. For example, in [58], comparing the effect of reinforcing a carbon fiber sheet and a steel plate, it is concluded that the carbon fiber sheet has excellent characteristics in terms of strengthening the structure. In [59], after studying the static load test of 19 beams reinforced with carbon polymer composite sheets, it is concluded that the direction of gluing carbon polymer composite sheets has a great influence on the failure mode of the beams. Currently, textile-reinforced concrete (TRC) has been developed—a composite material consisting of high-strength fine-grained concrete and corrosion-resistant textile reinforcement. This type of concrete has a bearing capacity that is essential for thin-walled structures [53]. Due to the corrosion resistance of textile materials, the thick concrete coverings known in conventional reinforced concrete are no longer needed. Thin new concrete elements are expanding the application of concrete to entirely new areas and giving architects and engineers more design options [54].

We have already considered variotropic (heterogeneous in the cross section) centrifuged and vibrocentrifuged reinforced concrete products and structures and have also proved their high efficiency [63,64,65,66,67]. At the same time, the behavior of a variotropic concrete structure reinforced with polymer composite rods and their influence on the operation of the structure remains unexplored [68,69,70,71,72,73,74]. In this regard, a promising direction is the development of new types of variotropic structures, particularly with the transition from annular sections to standard rectangular sections used for bending elements. Reinforced concrete beams and lintels, for the most part, work on bending tensile loads, and, of course, reinforcement plays a big role here. Thus, within the framework of scientific developments, we set the goal of the work—numerical justification using FEM modeling of the mutual work of concrete and polymer composite reinforcement and substantiation of the possibility of obtaining improved reinforced short bending elements from concrete with a variotropic structure. The main task and novelty are to investigate the influence of not only reinforcing elements but also the characteristics of non-homogeneous concrete. In view of significant differences in the work of concrete in different layers of bent elements, we have identified subtasks: firstly, to investigate the feasibility of the variability of the rectangular section of the bent element; secondly, to test the effects obtained in practice in the operation of such elements with the fixation of its operational reliability indicators, as well as the transition from reinforcement with steel rods to reinforcement with polymer composite rods. The relevance of the study is due to the global trend in the calculation and design of building structures aimed at improving the structural solutions of the elements, reducing the working sections, lightening the structures and facilitating the gradual transition from traditional steel reinforcement to more advanced polymer composite rods.

## 2. Materials and Methods

### 2.1. Materials

Three types of polymer composite reinforcement were used in the study: glass composite reinforcement manufactured by Kursk Plant of Composite Materials LLC (Kursk, Russia)-GCR; basalt composite rebar manufactured by VZMK LLC (Voronezh, Russia)-BCR; carbon composite fittings manufactured by Snabtekhmet LLC (Chelyabinsk, Russia)-CCR.

The main physical and mechanical properties of polymer composite reinforcement (PCR) are presented in Table 1.

### 2.2. Beam Design

As the basic object of study, jumpers were selected with the following dimensions: height 90 mm; width 120 mm; length 1030 mm. Heavy concrete of experimental lintel beams was used (class B30, bar reinforcement, 3Ø6).

The calculation was carried out based on SP 52-101-2003 [75], SP 63.13330.2018 [76] and ANSYS software. For a comprehensive analysis of the features of the work of variotropic bending elements reinforced with polymer composite reinforcement, we performed their numerical simulation using the finite element method in the implementation of the ANSYS software package. The calculation is performed in physically linear (elastic setting) and physically nonlinear (elastic-plastic setting with cracking) forms.

**Figure 1:**
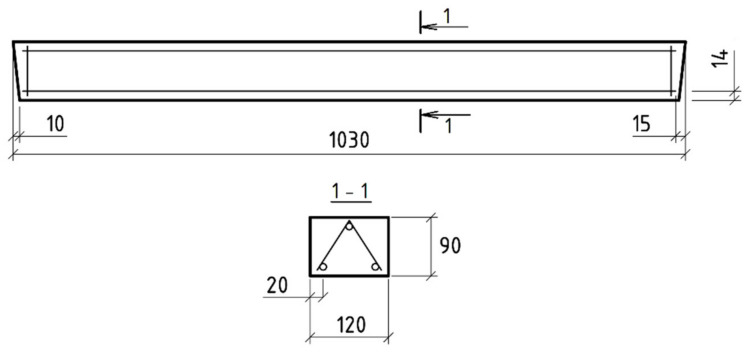
Geometric dimensions of lintel beams and the reinforcement scheme.

The geometric dimensions of small lintel beams and the reinforcement scheme are shown in Figure 1.

The production of products with a variotropic structure was achieved by placing an anode (anode zone) in the molded product in the zone of greatest strength and a cathode (cathode zone) in the zone of smallest strength, and a direct current was passed between them at a voltage gradient across the cross section of 0.5–5.0 V/cm V within 1–3 h from the moment of molding. After that, heat and moisture treatment were performed, or the products were kept until the concrete acquired tempering strength [77].

Subsequently, the author proposed an improved method for the manufacture of concrete and reinforced concrete products, which differs from [77] in that, to increase the differentiation of strength over the cross section of the product, the concrete mixture was preheated to a temperature of 30–90 °C [78].

We note in particular that, in the experiments, the integral characteristics of the concrete were both studied—generalized over the cross section—and differentiated—differing in terms of the cross section due to the different effects in the cathode and anode zones during activation.

### 2.3. Simulation Methods

To study the behavior of polymer composite (fiberglass, basalt composite and carbon composite) reinforcement reinforcing an inhomogeneous concrete beam, and to analyze their joint work, an FEM model was built in the ANSYS environment. The X-axis is directed along the longitudinal axis of the beam, the Y-axis is directed upward from the bottom face to the top and the Z-axis is directed perpendicular to the XY plane. Since the structure is symmetrical with respect to the OYZ plane, it makes sense to build half of the model.

**Figure 2:**
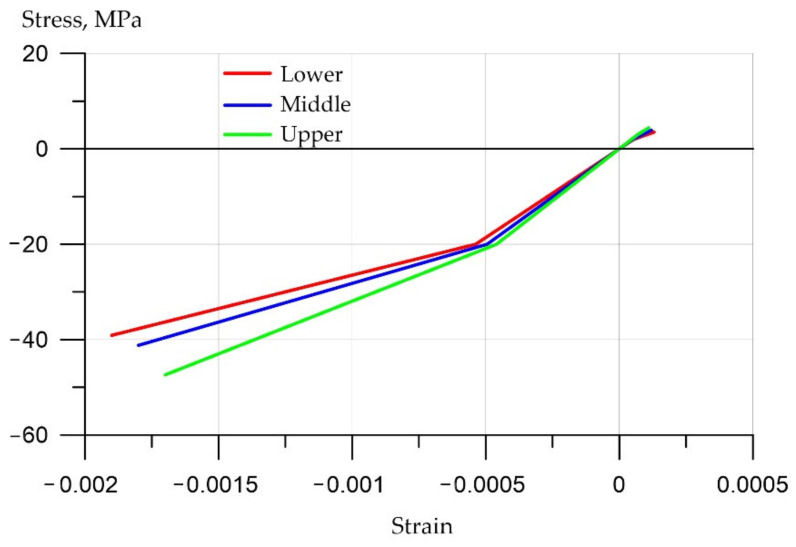
Bilinear stress-strain curves for the bottom, middle and top layers of concrete (negative stress values characterize compression).

The inhomogeneity over the cross section of the beam is represented by three layers (transversely isotropic medium), the properties of which are presented in Table 2. The elastoplastic properties of each layer have a bilinear stress-strain curve with isotropic hardening (Figure 2). The BISO option was used for the von Mises yield criteria with an isotropic work hardening. In the process of building the model, the number of elements varied widely (from 255 to 16380). When modeling, the geometry of the element should approach the recommended cubic one. At various stages of debugging the program, the number of elements was doubled, and the results were compared with the previous step until the discrepancy became less than 2%. Final version: 5772 elements, of which 5616 were SOLID65 elements (concrete); and 6890 knots. A larger increase in the number of elements and nodes did not lead to a significant change in the calculation results, so we stopped at this mesh size. The geometry of the concrete mesh is rigidly connected with the geometry of the reinforcement.

**Figure 3:**
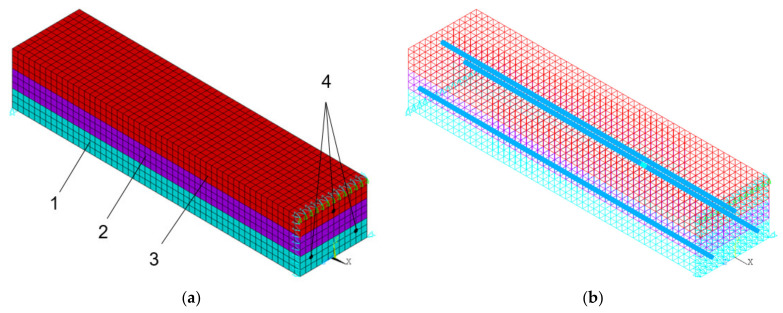
Beam model: (**a**) general scheme; (**b**) reinforcement layout; 1, 2, 3-layers of concrete; 4-reinforcing bars.

Concrete (layers 1, 2, 3) is represented by 8-node SOLID65 elastoplastic elements designed to model reinforced concrete with the possibility of cracking; reinforcing bars (element 4 in Figure 3) are modeled by BEAM188 cylindrical beam elastic elements tied to a grid of SOLID65 elements.

Concrete cracking was modeled according to the Willam–Warnke criterion implemented in ANSYS.

(1) $$\frac{F}{f_{c}}-S\geq 0$$

Here, *F* is a function of the principal stress state ($\sigma_{xp},\;\sigma_{yp},\;\sigma_{zp}$), *S* is a failure surface expressed in terms of principal stresses, *f_c_* = uniaxial crushing strength and $\sigma_{xp},\;\sigma_{yp},\;\sigma_{zp}$ are principal stresses in principal directions. The presence or absence of a crack is presented under the Willam–Warnke failure criterion based on the modification of the stress-strain relations by introducing a weakness in a direction normal to the crack face. If the material at an integration point fails in terms of uniaxial, biaxial or triaxial compression (or tension), the material is assumed to crush at that point. In SOLID65, crushing is defined as the complete deterioration of the structural integrity of the material. Under conditions where crushing has occurred, material strength is assumed to have degraded to an extent such that the contribution to the stiffness of an element at the integration point in question can be ignored.

For the analysis, three models were built:

1. Variotropic concrete reinforced with fiberglass rebar (GCR).
2. Variotropic concrete reinforced with basalt composite rebar (BCR).
3. Variotropic concrete reinforced with carbon composite rebar (CCR).

The mechanical characteristics of concrete and reinforcement layers are presented in Table 2.

Boundary conditions of the problem (Figure 3a,b):

1. All nodes with coordinate x = 0 are fixed in the direction of the Ox axis.
2. All nodes with coordinates x = −l/2; y = 0 are fixed in the direction of the Oy axis, where l is the length of the beam.
3. The node with coordinates x = 0; y = 0; z = −b/2 is fixed in the direction of the Oz axis, where b is the width of the beam.
4. All nodes with coordinates x = 0; y = h are connected in the direction of the Oy axis, where h is the height of the beam.
5. Force F = 7 kN is applied vertically down to the node with coordinates x = 0; y = h; z = b/2.

**Figure 4:**
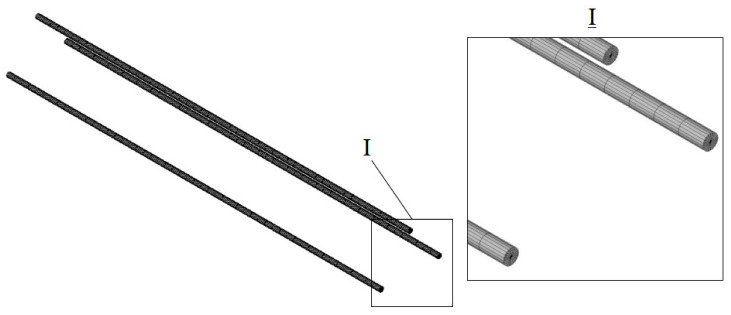
Reinforcement layout.

The scheme of the finite element partitioning of the polymer composite reinforcement is shown in Figure 4.

Table 2 presents the experimental data obtained in real tests carried out by the authors of [77,78]. Thus, the theoretical assumptions were verified according to experimental data in terms of concrete. Note that the data of the authors [77,78] are given for reinforced concrete bending elements with a differentiated, variotropic or transversally isotropic structure. We also performed a numerical simulation of transversely isotropic concrete in combination with a new generation of polymer composite reinforcement. Thus, in our work, we have shown a comparison of three cases:

(1) polymer composite-reinforced bending elements of the rectangular section;
(2) transversally isotropic bending-reinforced concrete elements;
(3) transversally isotropic polymer composite-reinforced bending elements.

Based on the experimental data of works [70,77,78], we carried out a numerical simulation of the third case and prepared the basis for the future experimental verification of physical work under the conditions of real experiments, which will be carried out by us in the future.

## 3. Results and Discussion

### 3.1. Numerical Simulation of the Operation of Variotropic Bending Elements Reinforced with Polymer Composite Reinforcement in Physically Linear and Nonlinear Formulations

**Figure 5:**
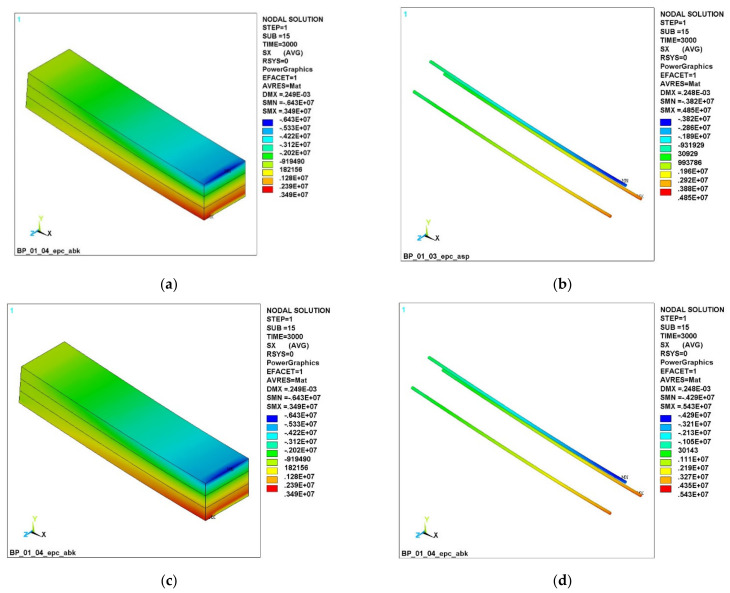

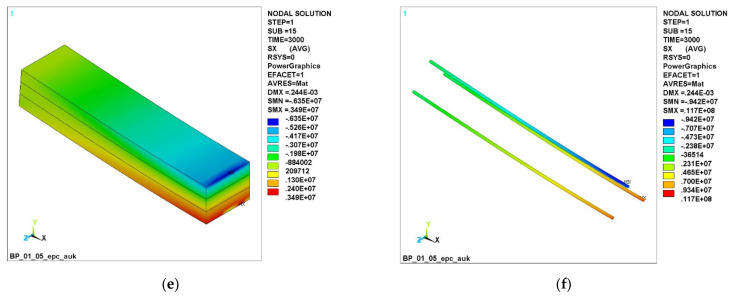
Normal stress fields $\sigma_{X}$ at a load of 3000 N: (**a**) in concrete with GCR; (**b**) in GCR; (**c**) in concrete with BCR; (**d**) in BCR; (**e**) in concrete with CCR; (**f**) in CCR.

The study of normal stress fields in concrete and reinforcement during loading shows that the moment of concrete cracking occurs at a load of about 3000 N. Until this moment, both reinforcement and concrete are loaded classically, with a uniform increase in stresses to their maximum values in the middle of the beam (3.42 MPa for concrete and 4.42 MPa for reinforcement). Figure 5 shows the stress fields at 3000 N for GCR, BCR and CCR reinforcement.

Figure 5 shows that, for CCR CFRP reinforcement, the cracking in the concrete started only in a small part of the lower chord (Figure 5e), while a beam with GCR (Figure 5c) and BCR (Figure 5d) cracks form throughout the lower part. A comparison of stresses $\sigma_{X}$ in polymer composite rebar (Figure 5b–e) shows that both the compressive and tensile stresses are higher in CCR bars, which is explained by a higher modulus of elasticity.

**Figure 6:**
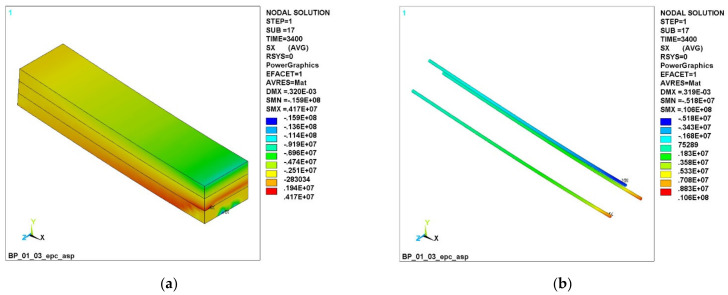

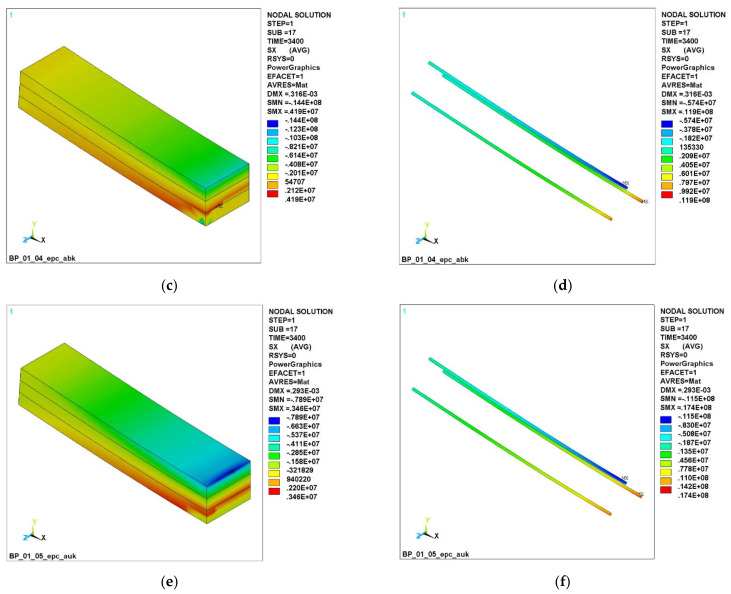
Normal stress $\sigma_{X}$ fields at a load of 3400 N: (**a**) in concrete with GCR; (**b**) in GCR; (**c**) in concrete with BCR; (**d**) in BCR; (**e**) in concrete with CCR; (**f**) in CCR.

Figure 6 shows the stress fields at a load of 3400 N.

It can be seen that, in the GCR beam (Figure 6a), cracks have reached the boundary of the lower and middle layers, and the green stress zone with the sign MN shows that the concrete has failed at the bottom. The beam with BCR is slightly better (Figure 6c); the cracking has passed through the boundary of the lower and middle layers, but the destruction has not yet occurred. In a beam with CCR, cracking did not reach the boundary of the lower and upper layers (Figure 6e); the CCR reinforcing bars work in an elastic deformation zone.

**Figure 7:**
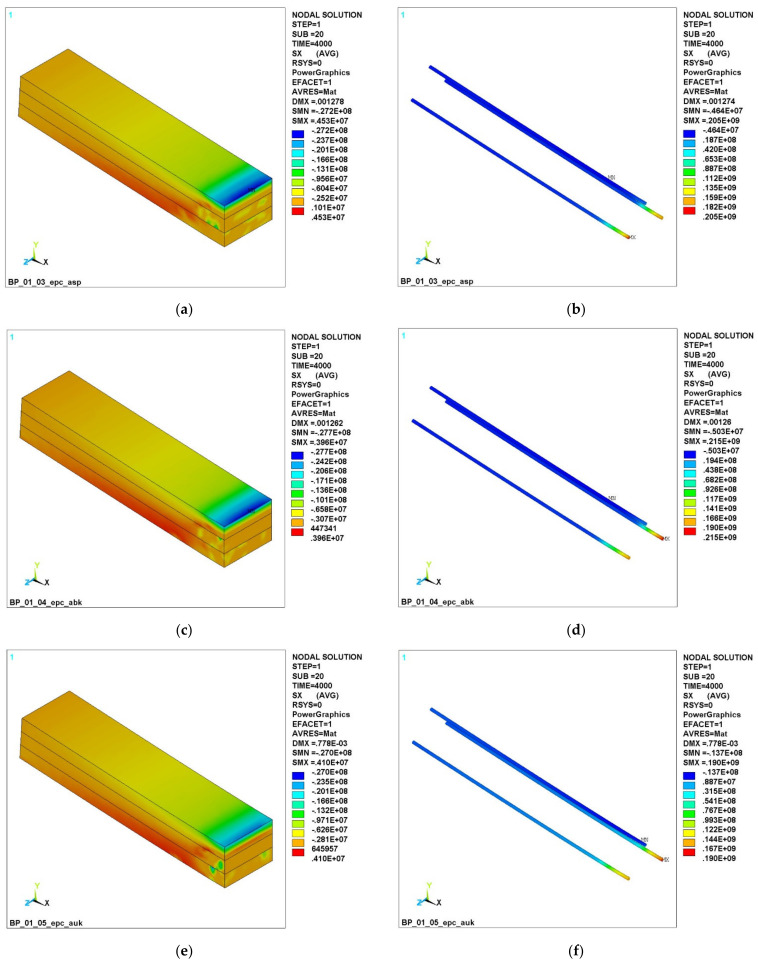
Fields of normal stresses $\sigma_{X}$ at a load of 4000 N: (**a**) in concrete with GCR; (**b**) in GCR; (**c**) in concrete with BCR; (**d**) in BCR; (**e**) in concrete with CCR; (**f**) in CCR.

Figure 7 shows the stress $\sigma_{X}$ fields at a load of 4000 N.

It can be seen (Figure 6a,c) that the beam with GCR and BCR failed, while the beam with CCR (Figure 6e) still resists bending in the top layer. The figure shows the fields of compressive stresses along the upper chord and the tensile stresses in the upper layer in the CCR zone of the reinforcement. It can also be seen that the tensile stresses in the GCR and BCR rebars (Figure 6b,d) increased sharply and exceeded the stresses in the CCR bars (Figure 6f). This is because the failure of the concrete has led to a situation where the entire load is taken only by the reinforcing bars, while, with carbon composite reinforcement (Figure 6f), the top layer of concrete still resists the load.

**Figure 8:**
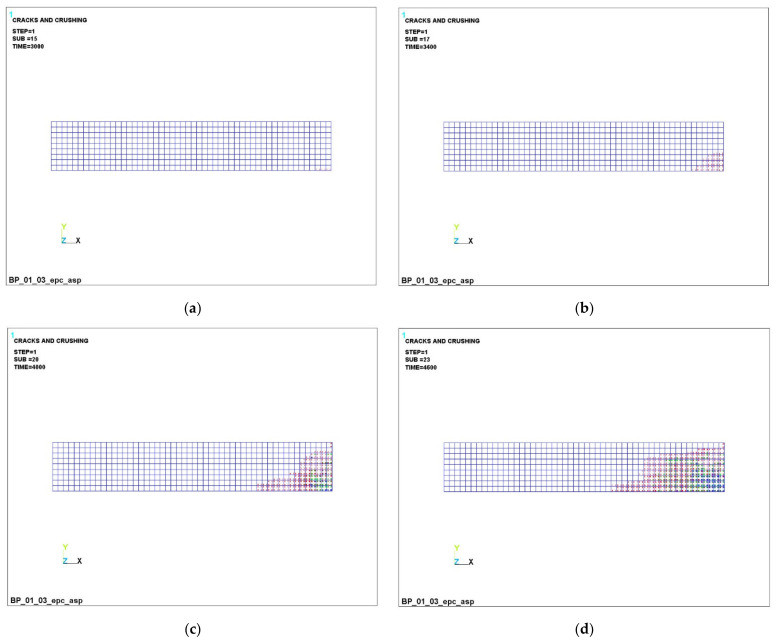
Successive cracking in concrete with GCR under load: (**a**) –3000 N; (**b**) –3400 H; (**c**) 4000 H; (**d**) –4600 H.

**Figure 9:**
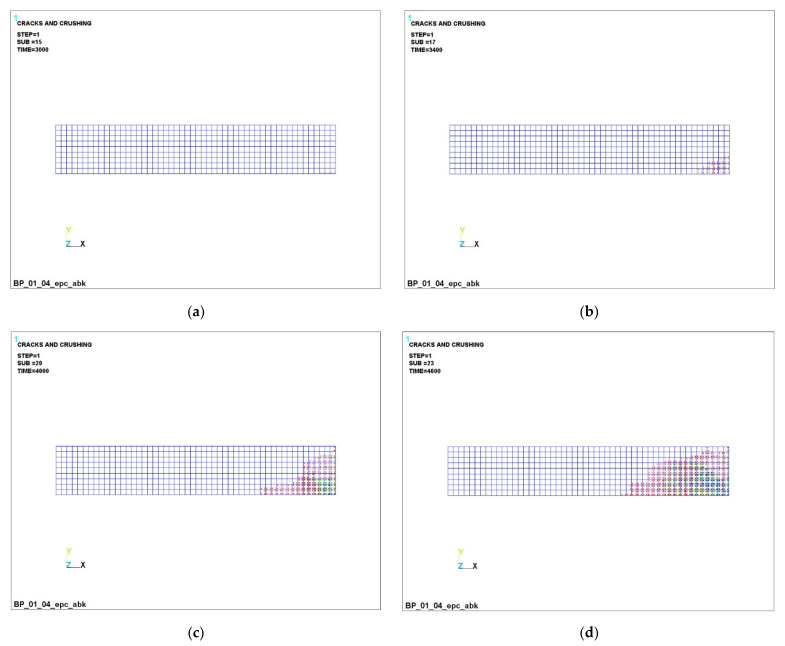
Successive cracking in concrete with BCR under load: (**a**) –3000 N; (**b**) –3400 H; (**c**) 4000 H; (**d**) –4600 H.

**Figure 10:**
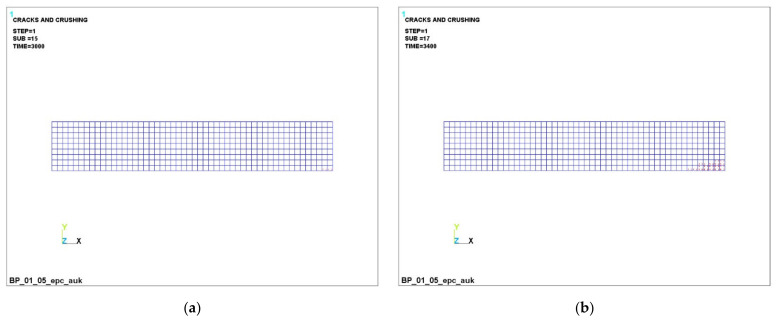

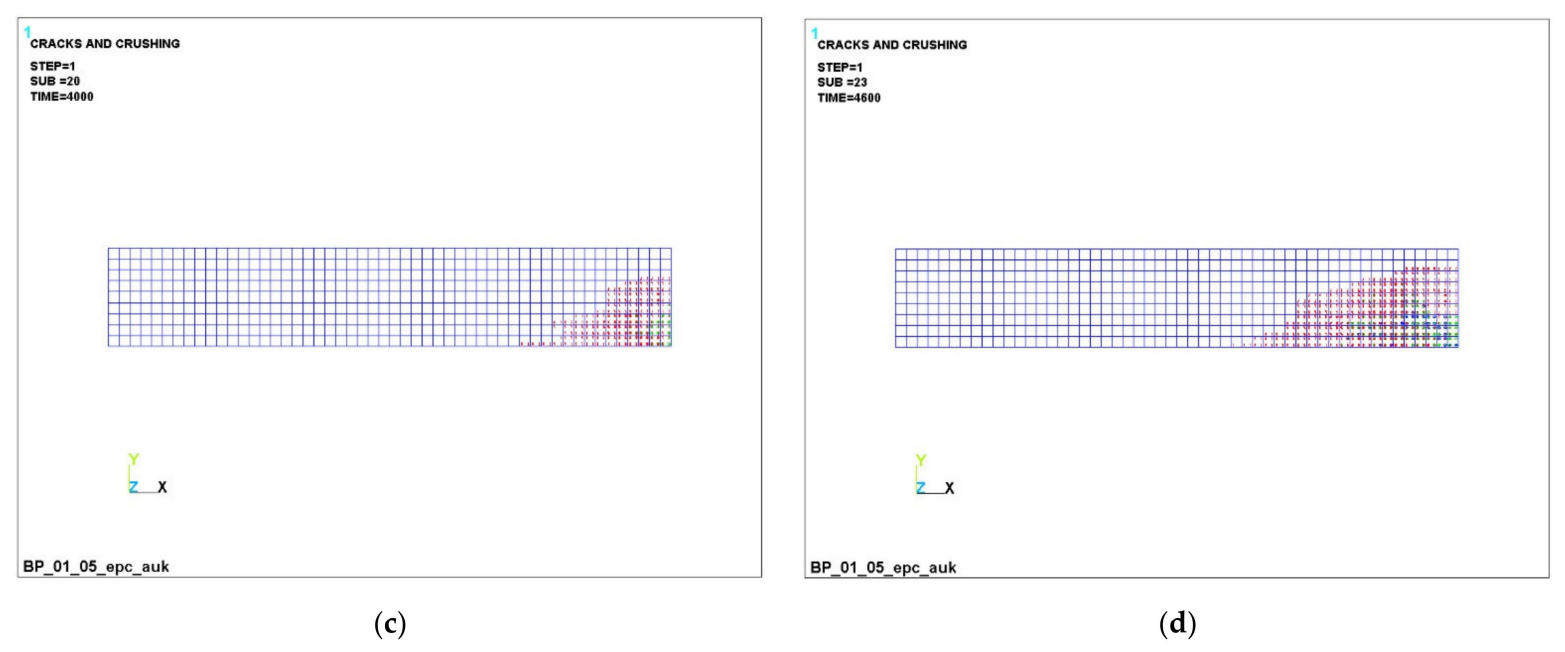
Successive cracking in concrete with CCR under load: (**a**) –3000 N; (**b**) –3400 H; (**c**) 4000 H; (**d**) –4600 H.

The successive development of cracks in a transversally isotropic beam is shown in Figure 8, Figure 9 and Figure 10.

It can be seen that, at a load of 3000 N (Figure 8a, Figure 9a and Figure 10a), concrete cracking begins in the lower part of the beam, which is accompanied by its unloading in this zone and an increase in the concentration stresses in the reinforcement, because it is she who begins to take on the entire tensile load. This process is clearly visible in Figure 5a,b (stresses in concrete are 3.47 MPa, in reinforcement −4.85 MPa).

A further increase in the load clearly reflects the abovementioned nature of the redistribution of stresses (Figure 6 and Figure 7). So, at a load of 4000 N, almost the entire lower part of the concrete in the dangerous section is destroyed, and the entire tensile load is taken up by the lower reinforcement. So, the maximum stresses in concrete are 3.87 MPa; in the reinforcement, it is already 484 MPa. At the same time, the nature of the loading of the reinforcement has significantly changed (Figure 7b,d,f).

The dynamics of the development of cracks in concrete can be seen in Figure 8, Figure 9 and Figure 10. In the figures, cracking is shown by the contour of a circle in the plane of the crack, and crushing is shown by the contour of an octahedron. If the crack opened and then closed, the outline of the circle will be marked with a cross. Each integration point can crack in three different planes. The first crack at the integration point is shown with a red circle outline, the second crack is shown with a green outline and the third crack is shown with a blue outline. An analysis of the development of cracks clearly shows the almost complete destruction of concrete in the dangerous section and nearby areas, which is fully confirmed by the increased load on the reinforcement in these places.

### 3.2. Discussion

The stress fields and the nature of the destruction of concrete for beams reinforced with basalt composite and carbon composite reinforcement have a similar nature; the differences are only in terms of numerical values. The analysis of the values is presented below.

**Figure 11:**
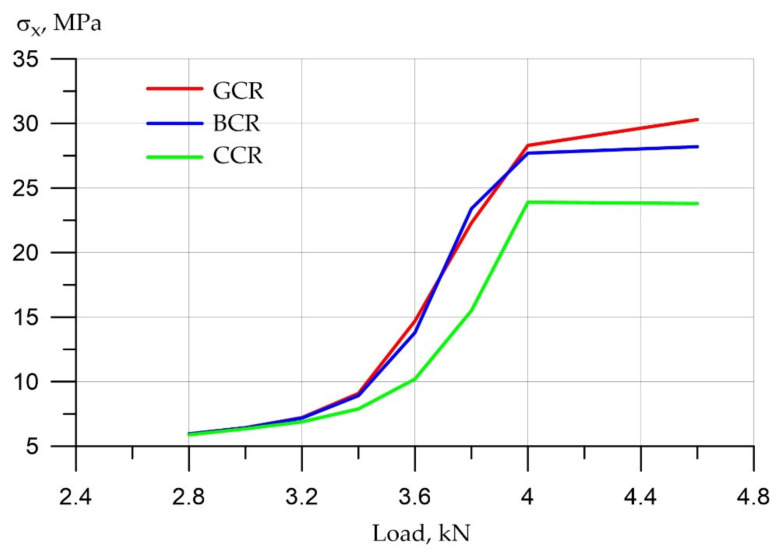
Compressive stresses in concrete with different types of reinforcement.

First, let us analyze the changes in compressive stresses in concrete (Figure 11).

By analyzing the graph of compressive stresses in concrete, it can be seen that the increase in stresses with increasing loads occurred according to a quadratic dependence from 2.8 to 4 kN. This is explained by the fact that the stresses grew both due to an increase in the load and due to a decrease in the beam section due to cracking. After active cracking by 4 kN and more—up to 7 kN—the increase in stresses occurs mainly due to an increase in the load, that is, due to one factor. Therefore, the growth graph acquires an almost linear dependence. It can be seen that the compressive stress in carbon fiber reinforcement is the smallest, that is, it is approximately 25–30% less than that of fiberglass and 20–25% less than that of basalt reinforcement.

**Figure 12:**
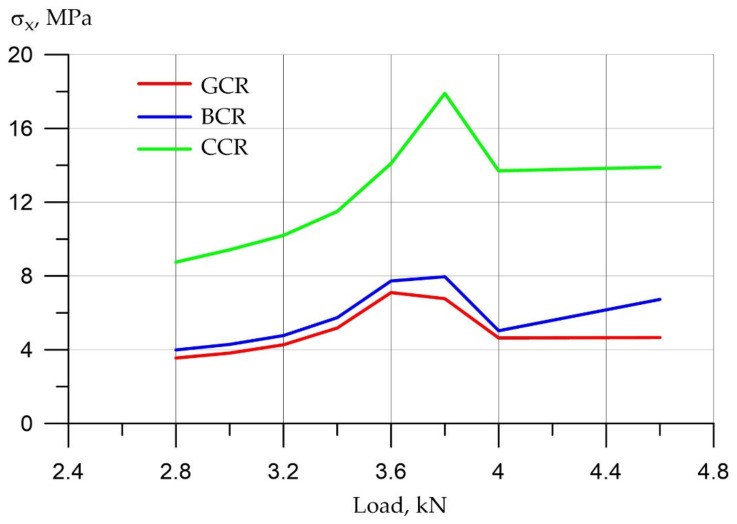
Compressive stresses in various types of reinforcement.

Next, we present a graphical interpretation and analysis of the results on compressive stresses in reinforcement (Figure 12).

When analyzing the graph of compressive stresses in the reinforcement, it can be seen that the ratio of stresses in the reinforcement correlates with the ratio of the deformation moduli of these materials from which the rods are made. As can be seen, more rigid carbon composite reinforcement takes on approximately 2.2–2.4 times greater compressive stresses than basalt and fiberglass. Basalt with fiberglass has very close values, which indicates approximately equal characteristics. It can be seen from the graph that, in the area from 3.5 to 4 kN, a surge of stresses is observed, after which their gradual linear decrease to the initial values occurs. Thus, we pay attention to the surge of stresses during the period of active cracking and the further redistribution of stresses to the compressed zone of concrete. Thus, by removing stresses from the reinforcing bars, the stresses are redistributed to the concrete.

**Figure 13:**
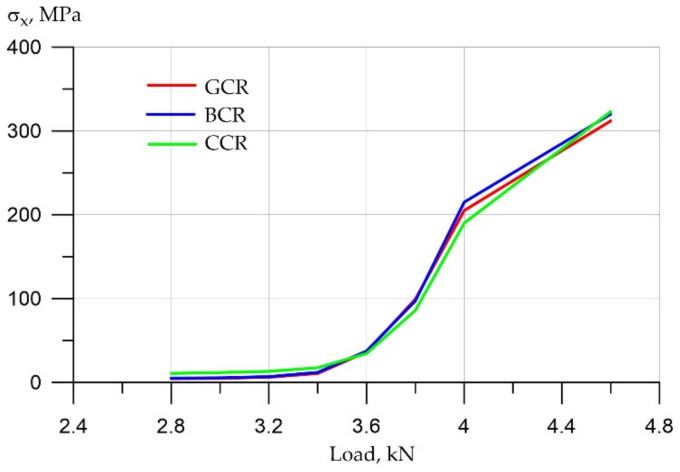
Tensile stresses in various types of reinforcement.

Consider and analyze the changes and the identified dependencies for tensile stresses in the reinforcement (Figure 13).

Before active cracking, the tensile stresses in the reinforcement, as well as the compressive ones, were proportional to the ratio of the deformation moduli, that is, the carbon composite reinforcement-assumed stresses that were two times higher than the stresses of fiberglass and basalt reinforcement. After 3.5 kN, after the start of active cracking, we find that there is an uncontrolled increase in stresses by two orders of magnitude, that is, hundreds of times. At the same time, we cannot clearly say which armature has more stress and which has less, that is, the graphs show heap, dense results. Accordingly, it can be seen here that the differences in work are relevant until the moment of active cracking.

**Figure 14:**
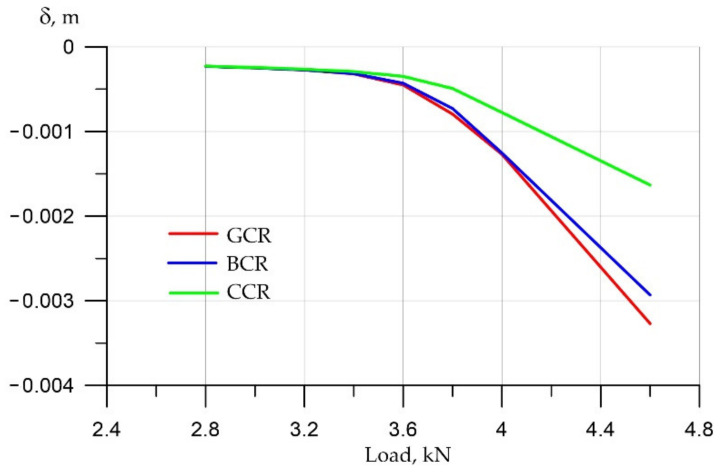
Dependence of beam deflections on applied loads.

Finally, let us analyze the results obtained for beam deflections (Figure 14).

The analysis shows that, again, in the zone of elastic work before cracking, we see linear dependencies, and after 3.6 kN, the deflections begin to grow more actively due to cracking. Since the cross section decreases due to cracks, the stress in the reinforcement begins to grow enough at this moment actively, and at the same time, we see a significant increase in deformations. According to the deformations, we see that deflections in the variant with carbon fiber reinforcement are also 2–2.5 times less than those with fiberglass and basalt composite reinforcement. This suggests that the rigidity of carbon fiber reinforcement is more than two times greater, respectively, with equal tensile stresses, which we described in the previous graph. It gives proportionally less deformation, that is, it is stiffer and deforms less.

**Figure 15:**
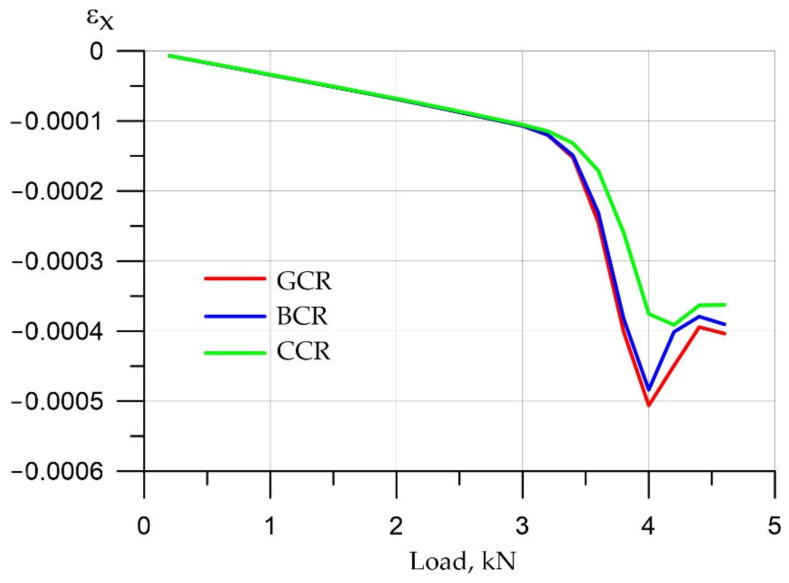
Concrete deformations in the compressed zone (top layer, top plane).

The graph of deformations in concrete is shown in Figure 15. One can see a nonlinear increase in the deformations in concrete and an abrupt change at a load of 4000 N. The decrease in deformations in the compressed region after 4000 N is due to significant cracking and a decrease in the effective section of the beam.

Summarizing all four previous graphs, we can summarize the following results.

For composite reinforcement, it is very important to take into account the work at the stage before active cracking. While it works quite predictably, stress deformations are easily determined, and there is no fluctuation. However, after active cracking, we see that there is a sharp increase in deformations and a sharp increase in stresses, both in concrete and in reinforcement.

Thus, we must clearly monitor the conditions for not exceeding the limiting moment for the onset of cracking in order to prevent uncontrolled processes in any case. However, at the same time, the manifestation of such a property as the formation of cracks in composite reinforcement is the weakest point, that is, it is the most critical parameter for them, since the issue of adhesion to the surrounding concrete due to winding is relevant, that is, the winding there is not as controlled as that in steel reinforcement, and the issue of small deformation moduli is also relevant. At the same time, we note carbon fiber reinforcement to be the one that works most adequately under conditions of active cracking; however, stresses there also grow quite strongly, and there is still a risk of uncontrolled deformations despite the fact that deformations occur two times less frequently than those of fiberglass.

It is confirmed that fiberglass and basalt rebar are very controversial materials. When they are used in design and construction, it is necessary to clearly monitor the process of cracking, the process of deformation and the process of stress growth.

Considering the significant novelty of this study in terms of a non-trivial approach to the manufacture of reinforced concrete products and structures, we will compare the results obtained by us with the results of other authors in two aspects. The first aspect is the integral comparison of the indicators of the received beams with the help of recipe-technological and design solutions proposed by us. Thus, a polymer composite-reinforced beam has advantages over a bent element of a similar section made using standard steel rods due to higher structural characteristics and the lower weight of the resulting structure, as well as the reduced cost. For example, due to the activation of concrete and giving it a variotropic structure, we reduced the deflections of short lintel beams with polymer composite reinforcement to 3%. This is in contrast to [3], where, due to activation by electric heating, the deflection of the bending elements increased. In [6], the deflection of reinforced concrete beams was regulated using additional holes in different places of solid and hollow elements. In this case, an increase in beam deflection was also observed [6]. An important factor of the study is the possibility, due to the proposed technology of imparting a variotropic structure, to reduce the deflection of bending elements with fiberglass reinforcement (glass composite and basalt composite), which, in turn, significantly increases the deflection in comparison with steel reinforcement [8,28].

In this regard, the results obtained by us in the design characteristics, which do not affect the change in concrete along the height of the variotropic section, compare favorably in the direction of acquiring advantages in relation to works such as [1,2,3,4,5,6]. Further, it should be noted that, in the aspect of comparing the approach to the differentiation of concrete over the section of the element, the work was performed for the first time and correlates only with studies [77,78], which were taken as the basis for numerical experiments. Thus, the physical experiment obtained by the authors [77,78] revealed the promise of using modern approaches, including the study of polymer composite reinforcement of concrete bending elements with a variotropic section, and the results turned out to be very effective.

Thus, we continued research [77,78] and developed in the direction of structures of a new type, which differ in terms of modern requirements. We justified the advantages by calculating and modeling the resulting structures. The prospect of future research is seen in carrying out full-scale tests with the reproduction of the technology proposed in [77,78] and, in a large-scale aspect, the continuation of our research already conducted on long products and then on long-span ones.

## 4. Conclusions

We theoretically substantiated and confirmed, by numerical simulation, the efficiency of the operation of variotropic bending elements reinforced with polymer composite reinforcement in physically linear and nonlinear formulations.

(1) Numerical models of lintel beams of non-variotropic and variotropic structures with various types of reinforcement, made in a spatial formulation by physically linear and non-linear elements, have been developed. The stress-strain state of the concrete of lintel beams and polymer composite reinforcing bars of all considered types was studied.
(2) It has been established that, in a physically nonlinear setting, when using the integral characteristics of concrete, the deformability (deflections) of the elements is greater than that when using the differential characteristics of concrete along the height of the section (up to 5%). This trend is similar for all bending elements, regardless of the type of polymer composite reinforcement used.
(3) In the upper, most compressed zone, an increase in the compressive strength of concrete is required, and in the tensile zone, concrete practically does not work. Concrete transfers all forces to the reinforcement in the central zone in the middle of the beam at the bottom edge. In this case, the effect of a clear redistribution of stresses is in favor of elements with a variotropic cross section in height. The compressed fibers in the uppermost zone are most susceptible to anodizing, i.e., the highest strength. The height of the compressed zone of concrete is in this zone, where the work of the structure is most effective.
(4) It has been established that, in variotropic bending elements, carbon fiber reinforcement works most adequately under conditions of active cracking; however, stresses there also grow quite strongly, and there is still a risk of uncontrolled deformations, despite the fact that deformations occur two times less frequently than those of fiberglass. It is confirmed that fiberglass and basalt rebar materials are very ambiguous. When they are used in design and construction, it is necessary to clearly monitor the process of cracking, the process of deformation and the process of stress growth.

The continuation of the work is planned in the direction of conducting experimental studies and comparing the results of the numerical simulation with the results obtained in laboratory or field conditions.

## Figures and Tables

**Table 1:** Main physical and mechanical properties of PCR.

Characteristics	Reinforcement Type		
	GCR	BCR	CCR
Tensile strength, MPa	1236	1350	1528
Tensile modulus, GPa	56	63	140
Compressive strength, MPa	523	500	979
Relative extension, %	1.6	1.7	1.3
The nature of the behavior under load (dependence “stress-strain”)	Linear		
Density, kg/m³	1943	1900	1993

**Table 2:** Mechanical characteristics of the model element.

Number	Title	Characteristics
1	Lower layer	Modulus of elasticity: 3.721 × 10^10^ Pa Poisson’s ratio: 0.2 Tensile elasticity: 2002.6 kPa Tensile strength: 3500.0 kPa Limit relative deformation tensile: 0.00013 Compressive elasticity: 20,000 kPa Compressive strength: 39,100 kPa Limit relative deformation for compression: 0.0019 Tensile hardening modulus: 1.966 × 10^10^ Pa Compressive hardening modulus: 1.402 × 10^10^ Pa
2	Middle layer	Modulus of elasticity: 4.0408 × 10^10^ Pa Poisson’s ratio: 0.2 Tensile elasticity: 2002.6 kPa Tensile strength: 3900.0 kPa Limit relative deformation tensile: 0.00012 Compressive elasticity: 20,000 kPa Compressive strength: 41,200 kPa Limit relative deformation for compression: 0.0018 Tensile hardening modulus: 2.69 × 10^10^ Pa Compressive hardening modulus: 1.62 × 10^10^ Pa
3	Upper layer	Modulus of elasticity: 4.3508 × 10^10^ Pa Poisson’s ratio: 0.2 Tensile elasticity: 3002.6 kPa Tensile strength: 4400.0 kPa Limit relative deformation tensile: 0.00011 Compressive elasticity: 20,000 kPa Compressive strength: 47,400 kPa Limit relative deformation for compression: 0.0017 Tensile hardening modulus: 3.41 × 10^10^ Pa Compressive hardening modulus: 2.21 × 10^10^ Pa
4	Glass composite (GCR)	Modulus of elasticity: 5.6 × 10^10^ Pa Poisson’s ratio: 0.32
4	Basalt composite (BCR)	Modulus of elasticity: 6.3 × 10^10^ Pa Poisson’s ratio: 0.32
4	Carbon composite (CCR)	Modulus of elasticity: 1.4 × 10^11^ Pa Poisson’s ratio: 0.32

## Data Availability

The study did not report any data.
